# A chromatographic approach for investigating the proliferation ability of native *Saccharomyces cerevisiae* yeast strains under varying temperatures and ethanol concentrations

**DOI:** 10.3389/ffunb.2025.1542167

**Published:** 2025-05-15

**Authors:** Lambros Farmakis, Panailla Aslanidou, Lia Katsou, Nikoketa Moraiti

**Affiliations:** Laboratory of Physicochemistry, Instrumental Analysis and (Bio)Chemistry of Foods, Department of Food Science and Technology, University of the Peloponnese, Kalamata, Greece

**Keywords:** native yeasts, indigenous yeasts, yeast growth, ethanol tolerance, asymmetrical flow field flow fractionation

## Abstract

Native yeast strains have been proved to be of great importance for food industry. In the present work, two different *Saccharomyces cerevisiae* native yeast strains isolated from the must from *Moschofilero* and *Agiorgitiko* varieties, respectively, were studied in order to estimate the influence of temperature and ethanol concentration on their proliferation ability via asymmetric flow field-flow fractionation (AsFlFFF) technique. The growth rate of the yeast strains, was directly linked to the biomass production under these conditions and was finally investigated via the ability of AsFlFFF to separate particles according to their size. The experimental results showed that the native yeast *Saccharomyces cerevisiae* from the must of the *Moschofilero* variety has an ideal growth temperature of 15°C in the absence of alcohol but exhibits low resistance to ethanol. In contrast, yeasts from the *Agiorgitiko* variety exhibit resistance to 10% v/v ethanol and remain active for a longer period of time. The ability of these strains to grow under these conditions is a strong indication that they can be used as starter cultures in winemaking to improve the organoleptic characteristics of the produced wines. Yeasts from *Moschofilero* are suitable for starting fermentation under normal conditions, while yeasts from *Agiorgitiko* can be used both as starter yeasts and in ethanol environments. This study shows also that the asymmetric flow field-flow fractionation technique can be successfully used to monitor yeast growth under different experimental conditions.

## 1 Introduction

The influence of *Saccharomyces cerevisiae* yeast strains on alcoholic fermentation is of great importance for the composition, the sensory profile, and total quality of the final product (Bisson, 2004; Gerard et al., 2023).

Industrial strains of *S. cerevisiae* are genetically and physiologically different from lab-grown and wild strains due to years of adaptation to commercial settings. These strains respond to environmental changes, nutrient shortages, drying, and cold stress—factors that impact their ability to ferment sugar quickly and completely, produce more alcohol while tolerating high levels, create appealing flavors and aromas, clump together well for easier separation, efficiently break down complex sugars, and produce little foam—and are specially developed for commercial use (Johnson and Echavarri-Erasun, 2011).

Ethanol serves both as a natural metabolite of yeast and as a significant stressor, with a negative impact on their physiological activity and fermentation efficiency. Morphological changes, destabilization of cellular structures (including the cell membrane and wall), protein damage, mitochondrial dysfunction, and accumulation of reactive oxygen species can be attributed to exposure to ethanol stress (Yang et al., 2024). Yeast adaptation to ethanol involves hundreds of genes related to metabolism, transport, cell structure, and biosynthesis pathways (Ma and Liu, 2010).

In recent years, the international scientific community has shown increased attention to native (or indigenous) yeasts as their influence on both the chemical composition and sensory profile of wine has proved to be of great importance (Lappa et al., 2020; Ge et al., 2023; Zhang et al., 2024). Their impact on these characteristics is due to their role during fermentation or because of their contribution prior to harvest through biological control of the root-knot nematodes of grapevines and fungal pathogens causing Botrytis bunch rot and black sour rot (Wanga et al., 2020; Hu et al., 2024). The influence of native yeasts on winemaking has been studied in wines of different geographical origins such as California, Texas, Brazil, China, Germany, New Zealand, and South Africa (Gayevskiy and Goddard, 2012; Bokulich et al., 2014; de Ponzzes-Gomes et al., 2014; Brysch-Herzberg and Seidel, 2015; Setati et al., 2015; Vigentini et al., 2016; Li et al., 2018; Bougreau et al., 2019), as well as for various varieties, such as Cabernet Sauvignon (Wang et al., 2021), Malvar (García et al., 2023), Malvazia/Vinos de Madrid (PDO) (Crespo et al., 2023), Merlot (Ut et al., 2022), Muscat and Tannat (de Ovalle et al., 2021), Riesling (Zoecklein et al., 1997), Sauvignon Blanc (Veloso et al., 2024), and Shiraz (Devi et al., 2022). Native yeasts have been also used for the reduction of ethanol in red wines (Maturanoa et al., 2019).

Native yeasts can also play an important role in other fermented beverages, such as agave-based drinks (Páez-Lerma et al., 2013; Nunez-Guerrero et al., 2016), Brazilian cachaça (Portugal et al., 2017), beer (Larroque et al., 2021; Moreira-Ramos et al., 2024), and coffee (Zhang et al., 2023).

Their applications have expanded to therapeutic uses (Kwak, 2024), preventing the impact of aflatoxins (Yahyapour et al., 2023) and ochratoxins (Meftaha et al., 2020), for their antifungal activity on ripe olive fruits (Pesce et al., 2018), their biological control of postharvest green mold in lemons (Pereyra et al., 2024), as starter cultures (Binati et al., 2019; Ooi et al., 2020), for biofuels and chemicals production (Kwak et al., 2019; Tsegu et al., 2022), for environmental applications (Ebrahimi et al., 2021; Vidal and Dávila, 2022), and for lipid production (Díaz-Navarrete et al., 2023).

The present study deals with native *Saccharomyces cerevisiae* yeast strains isolated from must derived from the *Moschofilero* and *Agiorgitiko* grape varieties cultivated at Mantineia and Nemea in the Peloponnese, respectively, which are among the most significant viticultural zones in Greece, producing Protected Designation of Origin (PDO) wines. Mantineia is considered the “birthplace” of *Moschofilero*, a noble, pink-skinned aromatic grape used to produce fine, dry white wines with intense floral and fruity notes (Nisiotou et al., 2019). *Agiorgitiko* is one of Greece’s major red grape varieties and is cultivated almost exclusively in the Nemea region, accounting for 5.8% of Greece’s total wine production (Ferrocino et al., 2025). To ensure terroir-driven wines that showcase the grape’s aromatic typicity, PDO production standards require that the grapes must be inoculated with selected starter yeast cultures (Nisiotou et al., 2019).

It is not a given that all strains of indigenous yeasts can function effectively under real fermentation conditions, such as increased alcohol levels and low temperatures. Therefore, in order for an indigenous yeast strain to be utilized in the industry, it must first be ensured that it can at least proliferate under these conditions.

This study aims to develop a fast and reliable methodology to determine the ability of yeast strains to multiply under high alcohol levels and fermentation temperatures. This approach will enable the selection of strains that can thrive under specific conditions so that only these strains are added to the fermentation process. Their impact on the qualitative characteristics of the final product can then be studied.

The proposed methodology is based on the ability of asymmetric flow field-flow fractionation (AsFlFFF) to separate particles according to their size. Alterations in the size of yeast cells can be directly linked to their growth and life cycle. Thus, the production or lack of biomass can serve as an indicator of yeast cell proliferation. Similar work combining the biomass growth with the activity of a microorganism during fermentation has already been published (Salazar et al., 2023). By monitoring biomass alterations, conclusions can be drawn regarding the yeast cells’ growth potential and so identify those strains with the potential ability to metabolize for further investigation.

For this purpose, yeast cell growth was investigated using the AsFlFFF (or AF4) technique, a sub-technique of field-flow fractionation (FFF), a high-resolution chromatographic method capable of separating and characterizing materials in the macromolecular and colloidal range based on their hydrodynamic size (Giddings, 1988). The AF4 is based on three basic steps: the sample injection; the sample focusing, where a counterflow—opposing the carrier solvent—is applied to concentrate all particles within a defined region before fractionation begins; and the fractionation, where the laminar flow moves the sample through the separation chamber. At the same time, the separation field is applied perpendicularly to the channel, counteracting the flow.

As particles travel through the channel, the cross-flow separation field pushes them toward the bottom. Simultaneously, Brownian motion causes them to diffuse back upward into the channel. The extent of this diffusion is size-dependent, and according to the size and the density of the sample granules, there are two different modes of separation: the normal mode, where smaller particles elute first since they exhibit greater Brownian motion and diffuse higher, while larger particles remain closer to the bottom; and the steric mode, where larger particles elute first since diffusion is greater than Brownian motion and smaller particles remain closer to the accumulation wall (Wahlund and Giddings, 1987; Giddings et al., 1991; Wang et al., 2020; Eskelin et al., 2021).

This property makes AsFlFFF particularly useful for this study, as yeast cell sizes change throughout their growth cycle. These changes can be assessed by measuring physicochemical parameters such as retention time and retention volume. Similar studies have been performed successfully in the past with another FFF sub-technique, sedimentation FFF (Farmakis and Koliadima, 2005; Farmakis et al., 2007). AsFlFFF is widely used as a separation technique with applications in biomedicine, biotechnology, colloid science, and environmental and life sciences (Baalousha et al., 2011; Yohannes et al., 2011; Wang et al., 2020; Plavchak et al., 2021).

## 2 Materials and methods

### 2.1 Preparation of the *S. cerevisiae* samples

The *S. cerevisiae* strains used in this study were provided by the research team of the project with MIS 5047289, which concerns the development of a scientific infrastructure for the study, conservation, and exploitation of the biodiversity of the microbial communities of the traditional fermented foods and wines of the Peloponnese region. The yeast strains were isolated from the must of *Moschofilero* and *Agiorgitiko* vine varieties. Each yeast sample was serially diluted and spread onto YPD agar media (1% w/v yeast extract, 2% w/v peptone, 2% w/v glucose, and 1.8% w/v bacteriological agar) (YPD, Condalab, Madrid, Spain). Next, the plates were incubated at 25°C –30°C for 2–5 days, allowing yeast colonies to develop. Morphologically distinct colonies were then sub-cultured on fresh media to obtain pure isolates (Suranská et al., 2016; Toyotome and Matsui, 2022).

Isolated strains were subjected to Matrix Assisted Laser Desorption Ionization – Time Of Flight Mass Spectrometry (MALDI-TOF MS) AutoflexIII (Bruker Daltonics, Bremen, Germany) analysis for identification using the default parameter settings within the MALDI Biotyper software ver. 3.1 (Bruker Daltonics, Bremen, Germany) (Holguín-Loya et al., 2025; Tsouggou et al., 2024). The *S. cerevisiae* strains, after they were isolated, were stored at -80°C (Skadi Green Line, model DF8517GLS). Next, having appropriately diluted the samples in saline solution, they were spread on YPD plates and incubated at 30°C for 48–72 *h* in a constant climate chamber (Memmert, HPP1060ECO). The colonies that appeared on the plates were isolated in the same medium. After ensuring purity, they were grown in YPD broth and stored at -20°C in 20% glycerol (Nuñez-Guerrero et al., 2019; Ut et al., 2022).

Furthermore, 50 μL of the *S. cerevisiae* sample was added to a sterilized testing tube containing 5 mL of YPD. The YPD had also been previously sterilized at 121°C for 16 min (model AES-75, Raypa Espinar, Barcelona, Spain). For further dispersion, the tube was put in a vortex mixer (model LVM – 202, LabTech, Italy). The procedure took place in a Laminar flow cabinet (model UCS 4, DesitGroup). The tube remained at 30°C for 2 days for the culture to grow.

### 2.2 Preparation of the *S. cerevisiae* cultivations

Since the study concerns the influence of temperature and alcohol concentration on the growth rate of the specific native yeast strains, the growth medium had a different composition each time. The adjustment of the alcohol concentration was performed by adding the appropriate amount of absolute ethanol (Fisher Scientific, UK, code: E/0665DF/17) into the growth medium after sterilization of the broth. For the development of the cultivations, conical flasks were used. Details are given in **Table 1**.

**Table 1:** Composition of the growth medium for different alcohol concentration.

Alcohol concentration (v/v)	YPD (g)	Ethanol (mL)	Triple distilled water (mL)
0	12.5	–	250.0
3	12.5	7.5	242.5
5	12.5	12.5	237.5
8	12.5	20.0	230.0
10	12.5	25.0	225.0

It is repeated for every temperature.

The flasks were sterilized at 121°C for 16 min. Each time, both the flask and the testing tube were under the same temperature for a few hours (7°C, 15°C, 20°C, and 30°C). Next, 2.5 mL of the *S. cerevisiae* sample from the tube was added to the flask. In order to avoid contamination, the procedure was always carried out next to a flame. For the adjustment of the temperature, a household refrigerator (7°C), double-walled glass connected to a water circulator (JULABO, model F12 coupled with Heating Immersion Circulator ED v.2) for 15 °C and 20 °C, and a heating oven (Memmert GmbH + Co. KG, Schwabach, Germany) for 30°C, were used.

### 2.3 AsFlFFF instrumentation and operation

**Figure 1:**
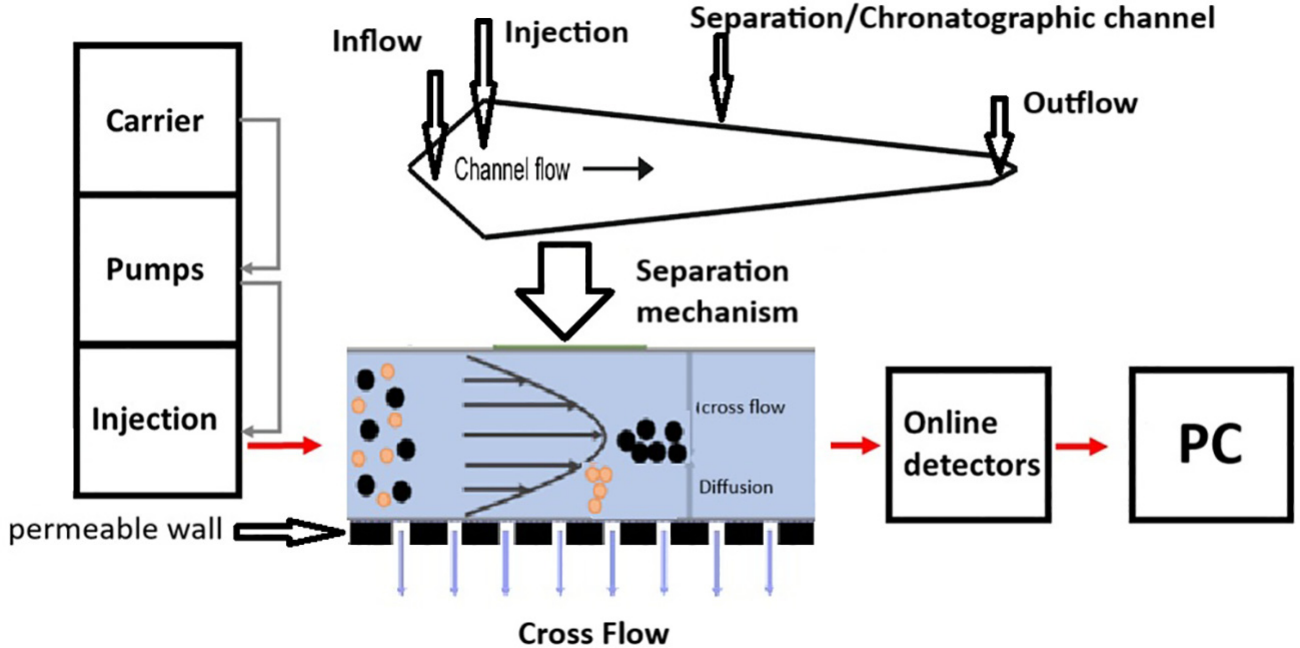
A schematic representation of an AsFlFFF system operating in steric mode.

In order to study the growth rate of the native yeast strains of *S. cerevisiae*, the Eclipse 3+ A4F separation system (Wyatt Technology Europe, Dernbach, Germany)—which is an AsFlFFF instrument—was used coupled with a Diode Array detector model PDA-M20A by Shimadzu. A schematic representation of an AsFlFFF system is given in **Figure 1**.

**Figure 2:**
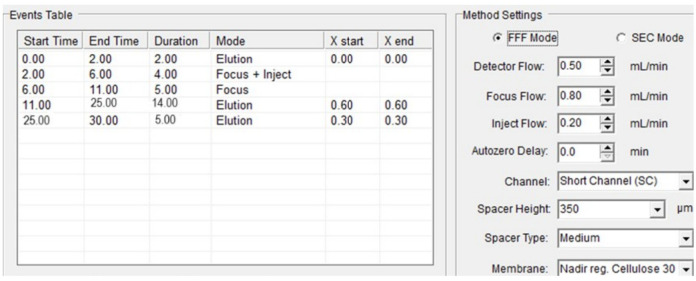
Experimental conditions for the AsFlFFF analysis.

An analytical channel 27 cm long with a 0.725 mL void volume, a 350 μm spacer made of Myler and PEEK, and a polyethersulfone Nadir Cellulose membrane with a molecular weight cut-off value of 30 kDa were used (Wyatt Technology Europe, Dernbach, Germany). The UV signal of the detector flow was monitored in volts (V) at 254 nm. The mobile phase was triple distilled water (3D H2O). The experimental conditions of the AsFlFFF system are given in **Figure 2**.

After the amount of the *S. cerevisiae* was added to the flask containing the growth medium, the flask was shaken gently for further dispersion. Next, 20 μL of the homogenized content of the flask was inserted into the AsFlFFF system manually by a micro syringe (SGE, Sigma-Aldrich) through a loop connected to a 4-port valve. The total experimental procedure and the adjustment of the experimental conditions described above were controlled through a PC using the Wyatt software program (Wyatt Eclipse, Shimadzu HPLC V1.0.12). The results were obtained on the PC in the form of chromatograms at 254 nm. The analyses of the chromatograms (retention time and volume, peak area, etc.) were also performed using the same software.

## 3 Results

### 3.1 Interpretation of a chromatogram

**Figure 3:**
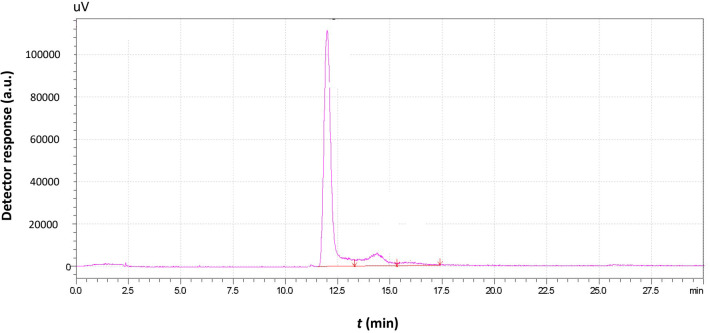
Typical chromatogram obtained by the analysis of a *Saccharomyces cerevisiae* sample with AsFlFFF also containing the data (e.g., area, peak) that can be obtained after integration of the chromatographic peaks.

A “raw” chromatogram obtained by AsFlFFF expresses the detector response profile over the separation retention time, as shown in **Figure 3**.

The separation of the sample granules is achieved due to the size distribution of the granules. In **Figure 3**, the existence of two different peaks indicates the presence of two different populations of *Moschofilero S. cerevisiae* yeast cells with different mean diameters. Since the size of the strains exceeds 1μm and their density ranges from 1.1068 to 1.1168 g mL^-1^—greater than that of the carrier liquid, which in this case was triple distilled water—it follows that the system operates under the steric mode of FFF. Consequently, the larger cells have a lower retention time and exit the chromatographic channel prior to the smaller cells (Wahlund and Giddings, 1987; Giddings et al., 1991; Wang et al., 2020; Eskelin et al., 2021).

The area of each peak is proportional to the number—and, correspondingly, the total mass—of the cells with retention times, within the peak limits. Thus, variation in either the retention time or the retention volume (area) of a peak corresponds to proportional variation in the mean size or total mass of the yeast cells, respectively. Since yeast cell growth is accompanied by changes in both the number and size of the cells, the yeast growth rate can also be correlated with variations in retention time and peak area over time.

### 3.2 Moschofilero

#### 3.2.1 In the absence of ethanol

As mentioned in the experimental section, *S. cerevisiae* native yeast strains were cultivated in media containing different ethanol concentrations (0%, 3%, 5%, 8%, and 10% v/v) while maintaining a constant temperature (7°C, 15°C, 20°C, and 30°C). A standard volume of each cultivation was injected into the AsFlFFF system, with injections repeated at specific time intervals until no further variations in the chromatographic peaks were observed.

**Figure 4:**
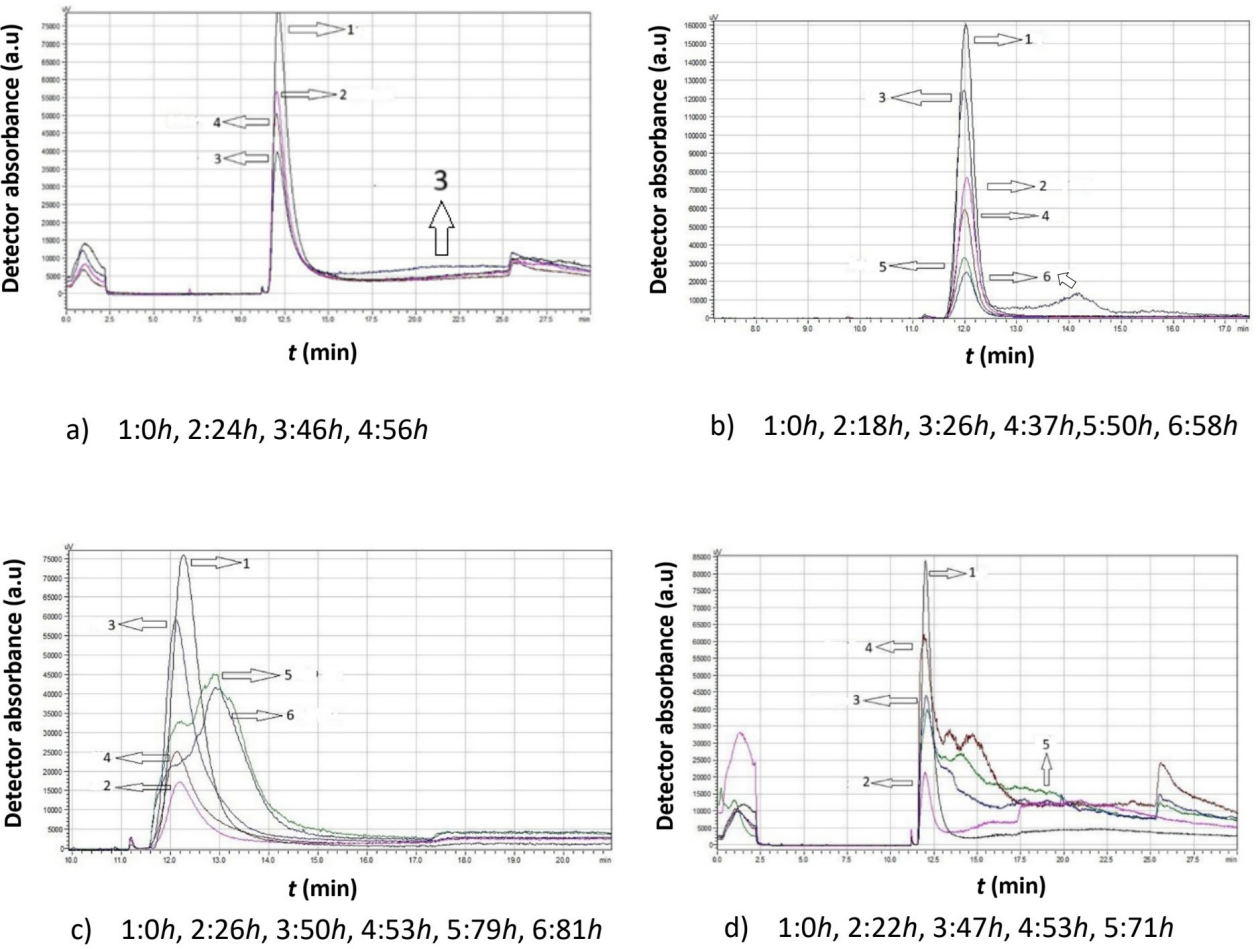
Data comparison of chromatograms obtained from *Moschofilero Saccharomyces cerevisiae* native yeast strain cultivations at different time intervals in the absence of ethanol at various temperatures: **(a)** 7°C, **(b)** 15°C, **(c)** 20°C, **(d)** 30°C.

**Figure 4** presents a comparative analysis of chromatograms obtained from *Moschofilero S. cerevisiae* native yeast strains cultivated in the absence of ethanol (0% v/v ethanol) at different temperatures. The selected chromatograms in each figure are representative of significant changes, providing a holistic view of the yeast cell behavior under these specific conditions.

The data in **Figure 4** clearly show that, at all temperatures, the highest peak was observed at the initial time point (zero hours), suggesting that the cells began dividing almost immediately.

At 7°C, a second peak corresponding to smaller cells appeared after 46 h. These small cells then consume culture medium and increase in size.

At 15°C, small cells were produced after 18 h and they continued to grow, leading to larger cells at 26 h. The peak height increased and shifted to the left. These cells continued dividing for several more hours, and after 58 h, a second peak appeared, indicating a significant ratio of small to large cells.

As the temperature increased, yeast cells exhibited distinctly different behavior. At 20°C, after almost 26 h, only small cells remained. Then, these cells grew again, forming new diploid cells and clusters (53 h). Eventually, a substantial number of small haploid cells were produced.

A similar pattern was observed at 30°C, where small haploid cells became predominant over time.

#### 3.2.2 In the presence of ethanol

**Figure 5:**
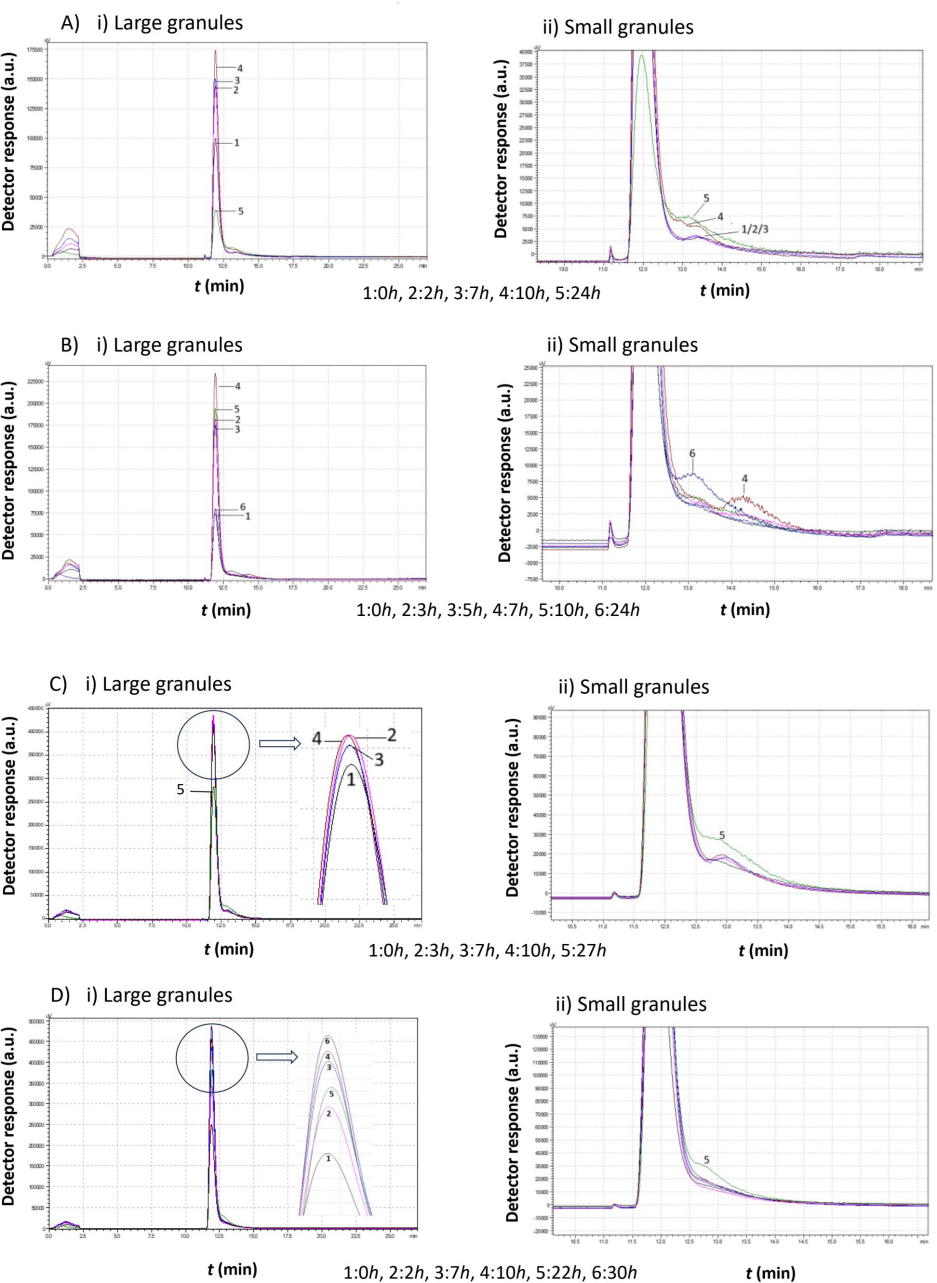
Comparison study of obtained chromatograms of *Saccharomyces cerevisiae* strains at 7°C with different ethanol concentrations, showing i) the first (large granules) and ii) the second (small granules) peak. **(A)** 3% v/v, **(B)** 5% v/v, **(C)** 8% v/v, and **(D)** 10% v/v at various times.

**Figure 6:**
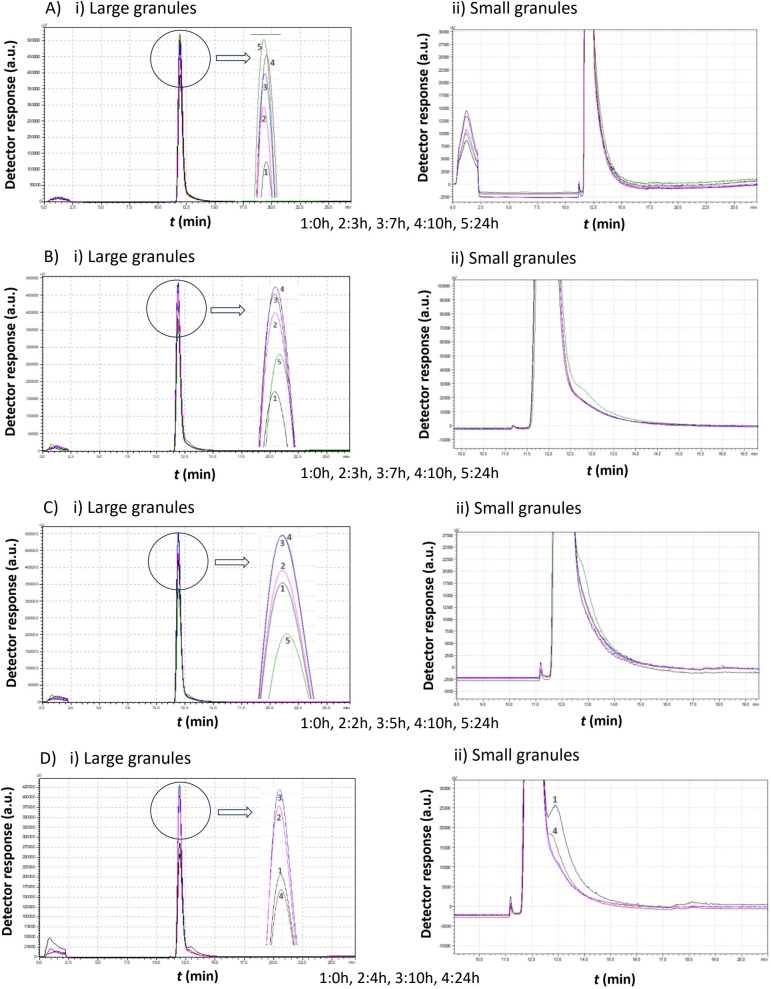
Comparison study of obtained chromatograms of *Saccharomyces cerevisiae* strains at 15°C with different ethanol concentrations, showing i) the first (large granules) and ii) the second (small granules) peak. **(A)** 3% v/v, **(B)** 5% v/v, **(C)** 8% v/v, and **(D)** 10% v/v at various times.

**Figure 7:**
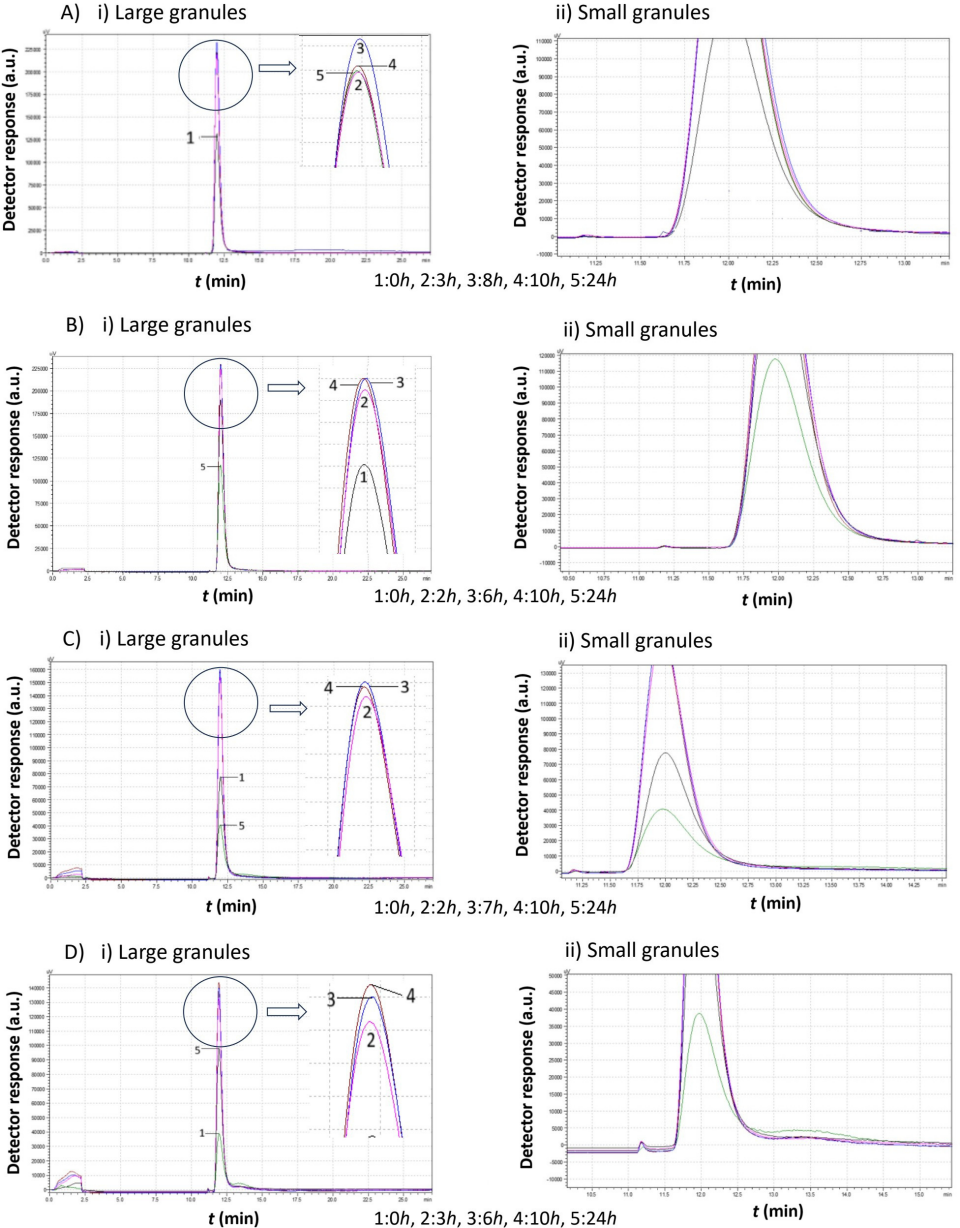
Comparison study of obtained chromatograms of *Saccharomyces cerevisiae* strains at 20°C with different ethanol concentrations, showing i) the first (large granules) and ii) the second (small granules) peak. **(A)** 3% v/v, **(B)** 5% v/v, **(C)** 8% v/v, and **(D)** 10% v/v at various times.

**Figure 8:**
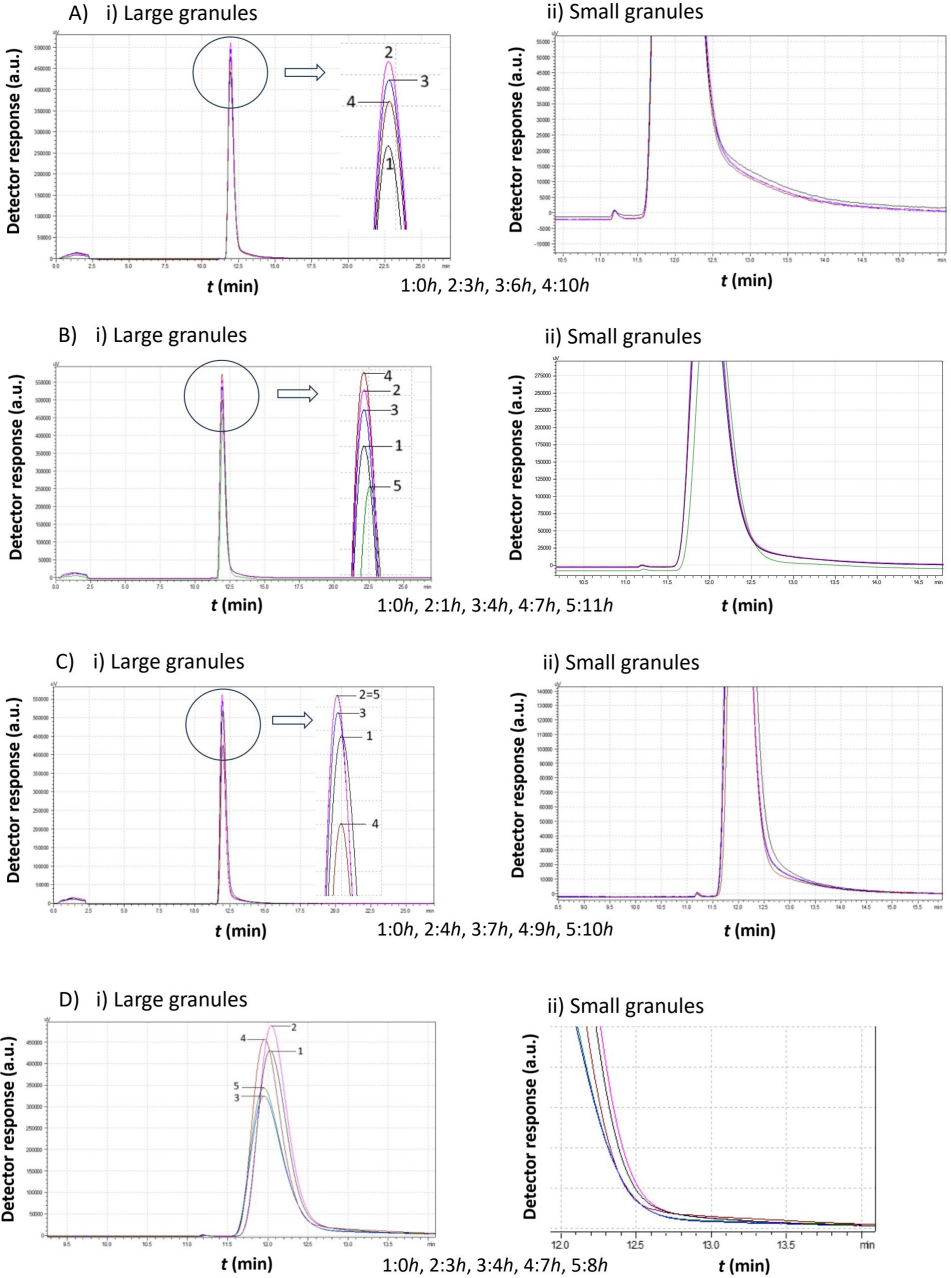
Comparison study of obtained chromatograms from *Saccharomyces cerevisiae* strains at 30°C with different ethanol concentrations, showing i) the first (large granules) and ii) the second (small granules) peak. **(A)** 3% v/v, **(B)** 5% v/v, **(C)** 8% v/v, and **(D)** 10% v/v at various times.

**Figures 5**–**8** illustrate the chromatograms obtained for *S. cerevisiae* strains under varying ethanol concentrations and temperatures. As previously mentioned, the chromatograms selected in each figure represent significant changes, offering a comprehensive picture of yeast cell behavior under these specific conditions.

At 3% v/v ethanol, the peak gradually increased over the first 10 h, while the mean cell diameter remained constant (same retention time). During this period, a second smaller peak emerged, corresponding to cells of a smaller size. After 10 h, the proportion of small cells increased, and by 24 h, the first peak declined sharply as more small cells were produced.

At 5% v/v ethanol, the peak increased during the first 7 h. Small granules then formed, leading to the appearance of a second peak with a higher retention time. The small cells continued dividing and, eventually, a significant proportion of small cells was observed. These cells had a larger mean diameter than those present at the 7 h mark.

At 8% v/v ethanol, the size distribution remained relatively constant, with both peaks maintaining similar heights. After approximately 27 h, a new population of cells with a lower mean diameter emerged.

At 10% v/v ethanol, a gradual increase was again observed over the first 10 h. The cells then divided, forming a second peak, which corresponds to smaller cells. These small cells consumed the remaining growth medium and increased in diameter. Notably, in contrast to lower ethanol concentrations, no small cells were detected before almost 20 h.

The data in **Figure 6** suggest that, in all cases, the production of small cells was minimal. However, this does not necessarily imply their absence.

In the first case (3% v/v), the peak shifted leftward after 3 h and then returned to the right at 10 h, reaching its final position by 24 h. This behavior indicates slight changes in cell size distribution over time.

At 5% v/v ethanol, the peak size increased over the first 10 h, indicating biomass production with a stable mean diameter (same retention time). Then, cell division occurred, the area of the peak reduced, and a small second peak appeared.

At 8% v/v ethanol, the same behavior was observed. The production of small cells was negligible.

Finally, at 10% v/v ethanol, a population of small cells was present at zero hours. These cells grew, increasing the first peak size. Eventually, these cells divided, leading to the formation of a population of small cells.

A key observation from **Figure 7** is the absence of the second peak corresponding to small cells in all cases. Regardless of ethanol concentration, yeast cells consumed the growth medium, leading to an increase in peak size. Although the peak area increased, which corresponds to a proportional increase of the total mass, the mean diameter remained constant. After almost 24 h, although the peak area decreased, no small cells appeared. The large cells probably cannot proliferate under these conditions, and since there was no more medium to consume, their sedimentation takes place.

Comparing the chromatograms of **Figure 8** with those of **Figure 7**, we found that *S. cerevisiae* native yeast strains from *Moschofilero* at 30°C show almost the same behavior as those at 20°C. In both cases, the second peak corresponding to small cells was completely absent. The main difference is that at 30°C, the total procedure lasted almost half the time than at 20°C.

For ethanol concentrations of 3% v/v and 5% v/v, cells consumed the growth medium and increased in mass over a few hours. They then divided into smaller cells, and the peak shifted slightly to the right, which means that the mean diameter was then a little lower.

At 8% v/v ethanol, it took almost 7 hours for the cell mass to increase. Smaller cells were produced after 9 h, as indicated by the higher retention time. Finally, these cells consumed the remaining growth medium and increased in mass and size.

When the ethanol concentration reached 10% v/v, the cells consumed the culture medium and increased their mass (3 hours). The total peak area decreased, which means a reduction in total mass, while the retention time also decreased, which indicates an increase in mean cell diameter (4 hours). The same phenomenon was repeated once more in a few hours. The cells consumed medium and increased their mass (7 hours) and then divided again (8 hours).

### 3.3 Agiorgitiko

#### 3.3.1 In the absence of ethanol

**Figure 9:**
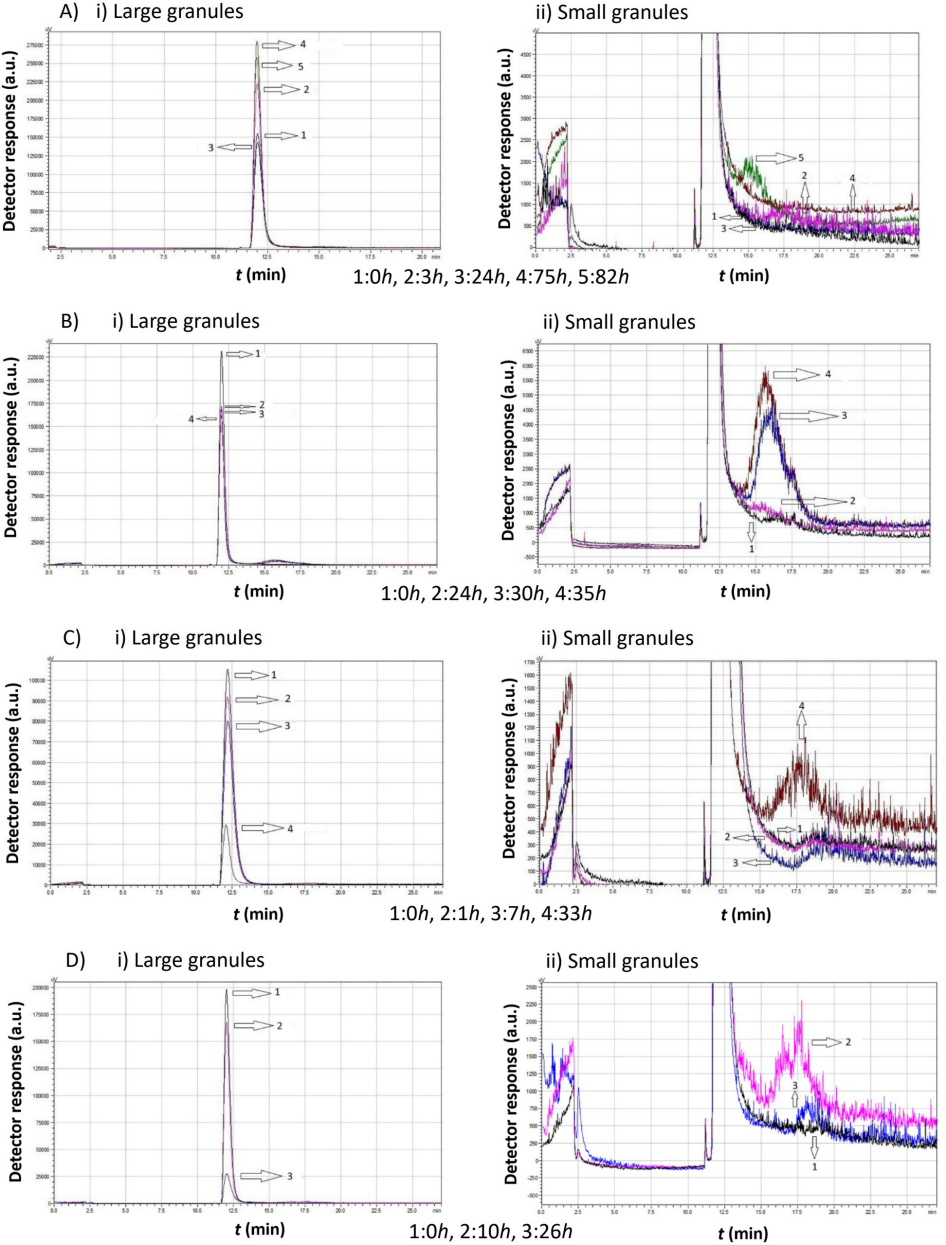
Data comparison of chromatograms obtained from *Agiorgitiko Saccharomyces cerevisiae* native yeast strain cultivations at different time intervals in the absence of ethanol at various temperatures, showing i) the first (large granules) and ii) the second (small granules) peak. **(A)** 7°C, **(B)** 15°C, **(C)** 20°C, **(D)** 30°C.

**Figure 9** presents a data comparison of selected chromatograms obtained from *Agiorgitiko S. cerevisiae* native yeast strains cultivated in the absence of ethanol (0% v/v ethanol) at different temperatures.

According to the data in **Figure 9**, when the *S. cerevisiae* cultivation was kept at 7°C, 3 h after inoculation, a significant increase of the first peak’s area (both in height and width) was observed compared to time 0. At the same time, there was a slight shift of the peak to the right, indicating that cell growth (in size and number) had begun with the simultaneous production of a few smaller cells, longer elution times, and a reduced mean cell diameter. At this point, a small second peak appeared, indicating the production of small-sized cells.

At 24 h, the sample shows almost the same behavior as at 0 h, indicating that the small particles had grown and the sample had entered an inert phase.

At 75 h, the area of the first peak increased significantly, reaching its maximum height and width. This suggests that the largest particles were at their maximum production rate and coexisted with double cells and clusters.

Finally, at 82 h, the area of the first peak decreased, while a significant production of small-sized cells occurred. The peak shifted further to the left, suggesting that the large cells had divided into new small cells, while cells in the form of clusters remained untouched. This was confirmed by the relatively large second peak, which corresponded to small-sized cells apparently produced by the proliferation of the original cells.

As the cultivation temperature increased, in the absence of ethanol, the yeast cells needed less time to reach their highest proportion of small cells. Thus, the second peak corresponding to the small cells was the highest at 34 h at 15°C, 33 h at 20°C, and only 10 h at 30°C.

#### 3.3.2 In the presence of ethanol

**Figure 10:**
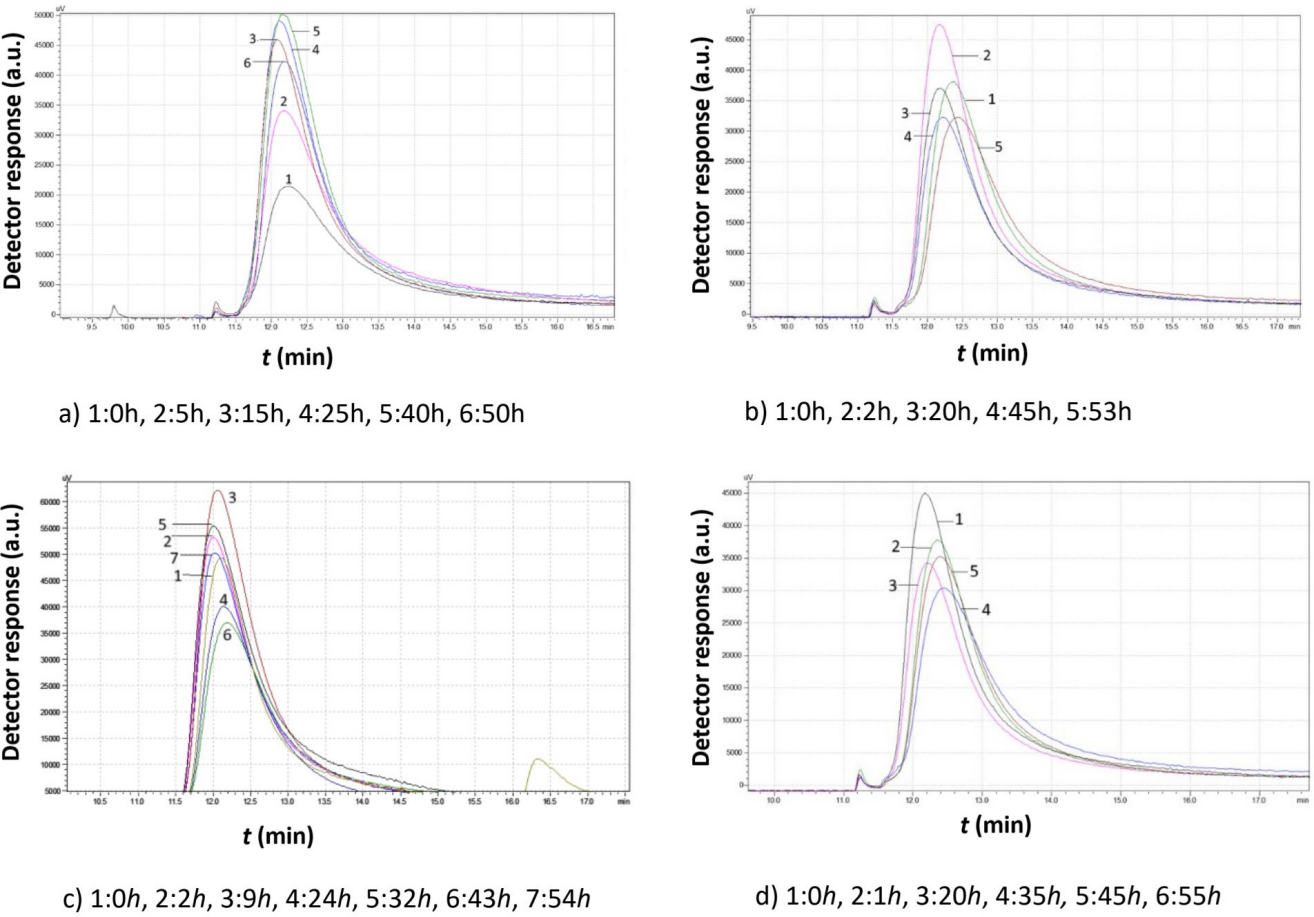
Comparison study of obtained chromatograms of *Saccharomyces cerevisiae* strains at 7°C with different ethanol concentrations: **(a)** 3% v/v, **(b)** 5% v/v, **(c)** 8% v/v, and **(d)** 10% v/v at various times.

**Figure 11:**
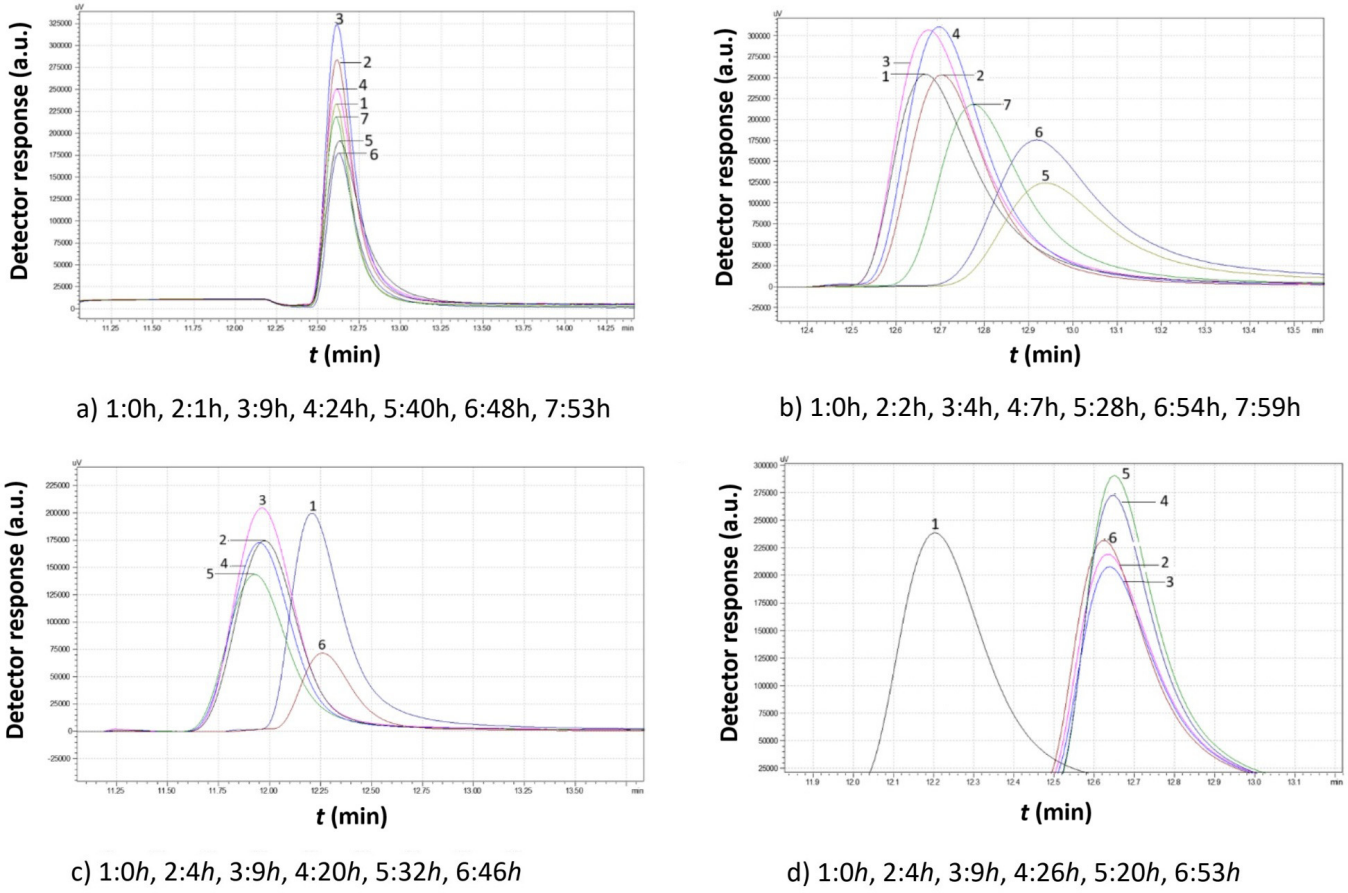
Comparison study of obtained chromatograms of *Saccharomyces cerevisiae* strains at 15°C with different ethanol concentrations: **(a)** 3% v/v, **(b)** 5% v/v, **(c)** 8% v/v, and **(d)** 10% v/v at various times.

**Figure 12:**
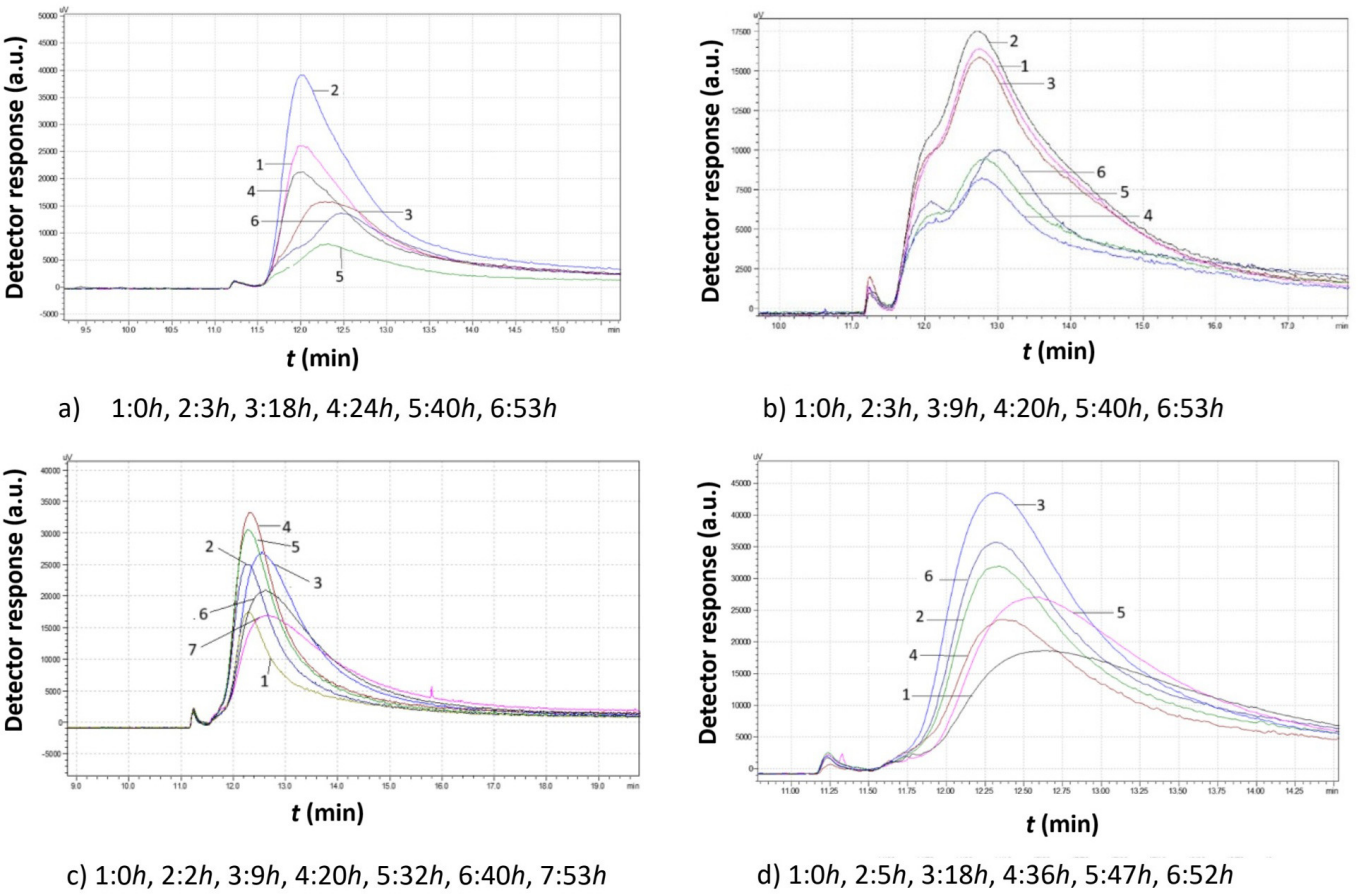
Comparison study of obtained chromatograms of *Saccharomyces cerevisiae* strains at 25°C with different ethanol concentrations: **(a)** 3% v/v, **(b)** 5% v/v, **(c)** 8% v/v, and **(d)** 10% v/v at various times.

**Figures 10**-**12** illustrate the chromatograms obtained for *S. cerevisiae* strains under varying ethanol concentrations and temperatures.

According to **Figure 10** (7°C), in the presence of 3% v/v ethanol, a continuous increase in peak volume was observed for approximately 40 h. The peak width also extended to the right, indicating the production of smaller yeast granules. After almost 50 h, the distribution of the grains remained constant. Initially, from 0 to 15 h, the peak shifted slightly to the left, suggesting that the granules consumed the growth medium and increased in size. After 15 h, the peak shifted slightly to the right as larger granules divided, reducing the mean size. At 5% v/v ethanol, the peak reached its maximum size within 2 h of inoculation. Rapid consumption of the medium led to an increase in strain volume. After 40 h, the strain volume decreased, stabilizing over the next 15 h as smaller granules were produced.

At 8% v/v, yeast granules grew for the first 9 h before producing smaller granules. This cycle repeated multiple times, indicating that under these experimental conditions, the yeast strains underwent more than one lifecycle.

When the ethanol concentration reached 10% v/v, the peak volume continuously declined, suggesting that the yeast strains were unable to proliferate under these conditions.

According to the data of **Figure 11** (15°C, 3% v/v ethanol), the peak increased for the first 9 h before gradually decreasing over the next 40 h. Finally, the remaining particles increased again until the growth medium was depleted.

In the presence of 5% v/v ethanol, the yeast granules began dividing almost immediately. Then, they grew for a couple of hours. For the next 20 hours, they multiplied, leading to smaller granules. The small granules consumed the remaining growth medium and increased in size again.

At 8% v/v ethanol, a rapid increase in mean granule size occurred in the first 4 h, as indicated by the lower retention time. Over the next 5 h, more granules were produced, leading to an increase in peak area. Finally, as the larger granules settled, a significant number of small granules emerged.

When the ethanol concentration reached 10% v/v, the yeast granules initially had a larger size due to the lower retention time. After 4 h, the peak area increased, but the retention time remained constant, indicating the production of small granules that did not grow further despite consuming the growth medium.

**Figure 12** shows the impact of ethanol on the proliferation of *S. cerevisiae* native yeast strains from *Agiorgitiko* at 25°C. In all cases, a broad chromatographic peak with significant tailing was observed, suggesting a wide distribution of granule sizes. Haploid cells (both small and large), diploid cells, and clusters appeared to coexist.

With 3% v/v ethanol, yeast cells proliferated significantly within the first 3 h. Then, the large cells divided into smaller ones, causing a rapid decline in peak area. Subsequently, the small cells grew again, forming larger cells (or diploid cells and clusters), which in turn generated more small cells.

At 5% v/v ethanol, the peaks became notably broad. For the first 10 h, yeast cells maintained a consistent size distribution. After that time, they divided into two populations with district mean sizes. Both populations consumed culture medium, increasing their total mass until depletion.

After increasing the ethanol concentration to 8% v/v, the yeast cells grew for the first 2 h before splitting into smaller cells. Then they consumed culture medium and increased in size. After approximately 30 h, they began to split again, forming smaller granules, as evidenced by shorter peaks with rightward tailing.

At 10% v/v ethanol, yeast cells grew continuously for almost 18 h. Over the next 18 h, smaller cells were produced, causing a rapid reduction in peak area. During the next 10 h, the peak became broader and shifted to the right, indicating the production of even smaller cells. Finally, as the remaining growth medium was consumed, the cells grew larger, leading to a higher peak that shifted to the left.

## 4 Discussion

The white varieties, *Assyrtiko*, *Malagousia*, *Moschofilero*, and *Roditis*, and the red varieties, *Agiorgitiko* and *Xinomavro*, are the most widespread varieties in Greece. The aromatic profiles of the wines have been determined and described in detail (Nanou et al., 2020).

The climate and soil of the vineyard, the variety, winemaking procedures, pre-fermentation processing, yeast strain, alcoholic fermentation conditions, and wine treatments from fining to bottling have been proven to be viticultural parameters that modulate wine aroma (Soufleros, 2015).

It has been demonstrated that diverse yeast species or strains may confer different characteristics to wines (Petruzzi et al., 2017; Sgouros et al., 2018). Thus, the use of yeasts derived from a vineyard may influence the typicity and genuineness of the respective local wines (Nisiotou et al., 2019 *).*

The combined use of commercial and native yeasts in winemaking has been proven to be an acceptable procedure to fulfill the growing demand for wines with a sense of the place of origin (Vaudour et al., 2015). Native yeasts have been used in order to preserve the typicity of wines. It has also been shown that indigenous *S. cerevisiae* strains strengthen the terroir expression in the resulting wines made from *Moschofilero* and *Agiorgitiko* varieties (Kontogiannatos et al., 2021). However, selected strains can only be used as starters in wine fermentation if the major characteristics of wine flavor remain essentially unchanged and if oenological parameters remain within acceptable limits (Romano et al., 2003).

In any case, native *S. cerevisiae* and *non-Saccharomyces* yeasts have been applied to wine fermentation and their influence on key oenological parameters, such as phenolic content, sulfur dioxide (SO2), ethanol tolerance, H2S production, pH value, total and volatile acidity, chromatic characteristics, volatile compounds, organic acids, and glycerol have been studied (Kogkou et al., 2017; Sgouros et al., 2018; Nisiotou et al., 2019; Kontogiannatos et al., 2021; Dimopoulou et al., 2022).

In previous studies, native yeasts were directly used as starters for wine fermentation, and their effects were determined. The present research focuses on one step before the addition of the yeast into the must and, for the first time, provides a chromatographic methodology for the monitoring of the ability of native yeast strains to proliferate under different conditions. It is important for wine makers to know how yeast strains react under specific fermentation conditions, as the use of native yeasts as starters can lead to final products with improved organoleptic characteristics only if these strains are ethanol tolerant and can proliferate under specific conditions.

This study aims to introduce a new chromatographic methodology for investigating the ability of *S. cerevisiae* native yeast strains to proliferate under varying temperatures and ethanol concentrations.

### 4.1 Moschofilero

#### 4.1.1 In the absence of ethanol

As the temperature increases, total activity of the yeast cells also increases, specifically from 51 h at 7°C to 81 h at 20°C. Then, there is a slight decrease to 71 h at 30 °C. At all temperatures, a distinct second peak of a relatively large area appears, indicating significant production of small particles, which can be attributed to a significant yeast proliferation and regeneration potential. Maximum particle production occurs at 20°C, suggesting that this temperature is probably the optimum for the proliferation of this particular yeast strain in the absence of ethanol.

#### 4.1.2 In the presence of ethanol

At 7°C, the total activity time is significantly reduced, ranging from 24 h at 3% v/v ethanol to 30 h at 10% v/v ethanol. As ethanol concentration increases, small particle production is reduced and almost eliminated. The elution times and, therefore, the average particle diameter, remain constant. This means that the production of small particles is limited.

The above demonstrates that, under these conditions, the ability of yeast cells of the specific strain to multiply is significantly restricted. This limitation is more pronounced with increasing ethanol concentrations.

At 15°C, the total yeast activity is around 24 h for all ethanol concentrations. At 3% v/v ethanol, peak size increases continuously, indicating that the yeast cells consume nutrient medium and increase in size but are unable to proliferate.

At higher ethanol concentrations, the peak size decreases, but no second peak appears. This possibly means that the larger clusters break down into smaller ones, some diploid particles divide, and, therefore, the total mass decreases, but the average diameter remains constant. In general, the ability of yeast cells to proliferate is limited.

At 20°C, the total yeast activity is also 24 h. At 3% v/v ethanol, the yeast cells increase in size in almost 3 h without the appearance of a second distinct population of smaller particles. As ethanol concentration increases, peak area decreases significantly after 10 h, possibly due to growth medium consumption and precipitation of the large aggregates. At 10% v/v ethanol, a small second peak appears, indicating both the formation of smaller particles due to disintegration and a low ability of the yeast cells of this particular strain to proliferate.

At 30°C, the total yeast activity is now limited to 10 h. At 3% v/v ethanol, yeast cells grow but do not proliferate. At 5% v/v ethanol, the peak decreases and shifts slightly to the right, indicating the formation of smaller particles (longer elution time). At 8% v/v ethanol, yeast cells seem to repeat their lifecycle, as the peak decreases noticeably, but almost immediately, the cells increase their size again until the growth medium is completely consumed. At 10% v/v ethanol, the cells complete two full lifecycles within just 8 h. However, over time, the peak shifts to the left, suggesting that large cell formation occurs. Essentially, it seems that with the consumption of the growth medium, no cell proliferation occurs, but most likely, the smaller particles grow, and the formed clusters settle due to their large size.

Overall, in the absence of ethanol, optimal yeast reproduction occurs at 20°C. The addition of ethanol inhibits activity, with the greatest inhibition of cell proliferation at relatively low ethanol concentrations (3% v/v). As ethanol concentration increases, the cells show increased activity but no proliferation. The cells increase in size until large clusters eventually precipitate.

### 4.2 Agiorgitiko

#### 4.2.1 In the absence of ethanol

As temperature increases, the yeast activity decreases significantly from 82 h at 7°C to 35 h at 15°C, 33 h at 20°C, and finally 26 h at 30°C. A bimodal size distribution of yeast cells was observed at all temperatures, with the highest small particle population at 15°C. At 7°C, the process occurs very slowly and the production of small particles is relatively low.

At 15°C, large particles gradually break down into smaller ones, indicating a high proliferation rate. At higher temperatures, cells consume nutrients, increase in size, and settle without proliferation.

#### 4.2.2 In the presence of ethanol

At 7°C, yeast activity is significantly reduced from 80 h in the absence of ethanol to 55 h in the presence of ethanol. Ethanol seems to completely eliminate the second peak, but the first peak becomes more flattened, which indicates a broad particle size distribution. The highest potential for cell proliferation appears at 8% v/v ethanol.

At 15°C and 3% v/v ethanol, the size distribution remains constant. Proliferation occurs at 5% v/v ethanol, while at 8% v/v ethanol, the cells increase in size and reach their original size distribution. At 10% v/v ethanol, the yeast cells divide, but only a limited production of small particles occurs. In any case, cells of this yeast strain appear to be tolerant to the presence of ethanol.

At 25°C, the yeast cells show consistent activity across all ethanol concentrations. At 3% v/v ethanol, the cells consume nutrient medium and increase in total mass while maintaining a constant average size. At 5% v/v ethanol, a bimodal size distribution appears, suggesting cell proliferation. At higher ethanol concentrations (8 and 10% v/v), peaks broadened to the right, suggesting polydispersity of the cells due to proliferation.

Differences in ethanol tolerance among these strains could be attributed to evolutionary adaptation events. It has been demonstrated (Voordeckers et al., 2015) that haploid cells, upon acquiring a mutation, became more capable of growth compared to the newly formed diploids. The resulting diploid cells exhibited significantly higher ethanol tolerance than the isogenic haploid strain. It was also observed that tetraploid cells (clusters) rapidly converted to a diploid state during the course of the evolutionary experiment. Additionally, some mutations were present for a short period and then disappeared, likely due to the dominance of another population (Vamvakas and Kapolos, 2020).

From the above, it becomes obvious that the size distribution of the yeast cells plays an important role in the ethanol tolerance of the different strains. Size distribution can easily be determined by AsFlFFF. This fact introduces AsFlFFF as a reliable technique for the kinetic study of cell proliferation.

In the present work, the AsFlFFF technique was used because the analysis can be achieved with low cost and in a short time. Since the chromatographic analysis obtained significant results concerning the ethanol tolerance of these *S. cerevisiae* native yeast strains, the gene sequence of these strains is planned to be determined. In this way, there could be potential differences detected in the genotype of these strains to which their different behavior to ethanol could be attributed.

Based on the above, it is shown that the AsFlFFF technique can be applied for monitoring the proliferation activity of yeast strains under different conditions. This succeeded due to the linking of the alterations of the size of the cells, and consequently the biomass production, with the growth rate of the yeast cells. Despite that, this technique cannot provide information about the ability of these yeast strains to metabolize, and further investigation is needed. It is important to note that the proposed methodology concerns the investigation of yeast strains grown in YPD broth and so any desirable experimental conditions can be applied in order to estimate the behavior of these strains in other fermentation procedures.

## 5 Conclusions

According to the results of this study, the asymmetric flow field-flow fractionation technique can be successfully applied to determine yeast cell proliferation under different experimental conditions.

The experimental results indicate that for *S. cerevisiae* native yeast strains isolated from the must of the *Moschofilero* variety, the optimum proliferation temperature in the absence of ethanol is 15°C. However, in the presence of ethanol, these native yeast strains seem to have negligible ethanol tolerance, and their overall period of activity is significantly reduced.

In contrast, the native yeast strains isolated from the *Agiorgitiko* variety seem to be ethanol-tolerant. They also remain active for a longer period in the presence of ethanol. From all of the above, it can be concluded that the *Moschofilero* native yeast strains can be used as starters in a regular fermentation procedure, while the *Agiorgitiko* native yeast strains can not only be used as starters but will remain active even if ethanol is produced.

## Data Availability

The raw data supporting the conclusions of this article will be made available by the authors, without undue reservation.
